# Nutritionally adequate food baskets optimised for cultural acceptability as basis for dietary guidelines for low-income Czech families

**DOI:** 10.1186/s12937-019-0510-y

**Published:** 2019-12-06

**Authors:** Kristyna Faksová, Zuzana Derflerová Brázdová, Aileen Robertson, Alexandr Parlesak

**Affiliations:** 1 Faculty of Health, Global Nutrition and Health, Institute for Nursing and Nutrition, University College Copenhagen, Sigurdsgade 26, 2200 Copenhagen, Denmark; 2 Department of Public Health, Faculty of Medicine, Masaryk University, Kamenice 753/5, 625 00 Brno, Czech Republic

**Keywords:** Nutritionally adequate diet, Linear programming, Cultural acceptability, Affordable diet, Low socio-economic status, Food-based dietary guidelines (FBDGs)

## Abstract

**Background:**

Czech nutrition recommendations prioritize health aspects without considering affordability. Low socio-economic groups have the highest risk of nutrition-related noncommunicable diseases and cost has been identified as an obstacle to achieve a healthy diet, making the implementation of affordability into dietary guidelines necessary. The aim of this study was to develop a food basket (FB) for a low income Czech family of four that is nutritionally adequate, health-promoting and culturally acceptable at an affordable price.

**Methods:**

Linear programming optimisation was used to ascertain that the FB covered the recommended nutrient intakes from the Czech Nutrition Society and from the World Health Organization (WHO). Cost of the FB was calculated on the basis of more than 3900 prices of 330 foods. Within a given cost constraint, all FBs were optimized for the highest possible similarity to the reported food group intake according to the most recent Czech National Food Consumption survey, which was used as a proxy for cultural acceptability.

**Results:**

The optimised FB affordable at a daily food budget for a Czech family on minimum wage (CZK 177, ~ € 6.8) contained 76 foods and had an average relative deviation of 10% per food category from reported intake. The main deviations were: 72% less sweets and confectionery; 66% less salt; 52% less meat; 50% less milk products; 8% less potatoes; and 484% more milk; 69% more oils and fats; 20% more cereals; and 6% more vegetables.

**Conclusions:**

The optimised FB can help to guide the development of food-based dietary guidelines for low income households in Czech Republic.

## Introduction

Noncommunicable diseases (NCDs) cause 90% of deaths in the Czech Republic, of which approximately 10 and 20% are preventable for men and women, respectively [1]. In 2017, the World Health Organization (WHO) estimated that approximately 25,600 lives could be saved if the country adopts WHO’s “best buys”, which are WHO’s recommended interventions for the prevention of NCDs and which include promotion of a healthy diet [2]. By the end of 2016, 19% of the Czech population were obese and 63 and 48% of Czech men and women were overweight [3], making the Czech Republic one of the leading European nations in this area [4]. Overweight and obesity increase the risk of developing diabetes mellitus type 2 (T2D) [5], the prevalence of which is rising particularly in men and which was associated with 2.2% of Czech deaths [6].

In the Czech Republic, only 9% of the population consumed the recommended minimum of five portions of fruit and vegetable per day while 46% consumed no vegetable or fruit daily [7]. Unhealthy eating habits, including high consumption of alcohol, saturated fat, salt and refined carbohydrates as well as a low consumption of vegetables and fruit, are among the most important behavioural risk factors for CVDs [8] and eating micronutrient-dense foods with low energy density can help prevent both noncommunicable diseases (NCDs) and micronutrient deficiencies [2, 9].

However, micronutrient-dense foods are relatively expensive [10] so especially low income families may buy less and increase the risk of malnutrition [11]. Indeed the prevalence of obesity in the Czech Republic is higher among those with low socio-economic status (SES) compared with high SES, similar to other EU countries [12]. Health inequalities in the Czech Republic are considered to be associated with obesity, T2D and high blood pressure [13–15]. Ultra-processed foods, sugars, and fats provide low cost energy sources and are designed to taste good, have a long shelf-life and are time-saving in contrast to nutrient-dense foods, such as raw lean meats, fish and vegetable [10] and may therefore be preferred by groups with a low socio-economic status. In 2016, the proportion of the Czech population at potential risk of poverty was estimated to be nearly 10 % (9.7%), based on an yearly income threshold of CZK 128287 [16].

The main goal of this study is to create a list of locally available foods (= food basket), which are nutritionally adequate, health-promoting, culturally acceptable and affordable for a low socio-economic status family of four in Czech Republic. Once created, this food basket (FB) could be considered as the basis for Czech food-based dietary guidelines (FBDGs), from where low income families get information to help reduce their health inequalities related to diet-associated non-communicable diseases. Moreover, the study investigates to which extent cultural acceptability, which is approximated by the similarity to observed food consumption patterns, is affected by the food budget.

## Materials and methods

### Budget available for food and non-alcoholic beverages of Czech families living on a minimum monthly wage and on a median income

From January 2018, the Czech minimum monthly wage per capita was CZK 12200 (€ 471), which provides a net income of CZK 10468 (€ 404) per month [17]. The median salary is CZK 27236, corresponding to CZK 20835 net (€ 1053 and € 805, respectively) [18]. The monthly child benefit was CZK 1442 (€ 55) per child and month [19].

Czech families with dependent children account for nearly one fifth (19.7%) of the household structures [20]. Therefore, the reference family for this study comprised a mother from the group aged 25–50 years, a father aged 35–39 years, a 16-year old daughter and a 7-year old son. This household composition allows for possible generalization to other low income families in Czech Republic.

The low income household budget available to buy food and non-alcoholic beverages was calculated by assuming that both adults earned the same minimum wage each, plus they received benefits for both children (CZK 1442 per child and month). According to the Czech Statistical Office, the first quintile (lowest 20%) of population with the lowest income spend on average 22.7% of their income on food and non-alcoholic beverages, while people in the third quintile (earning at least a median salary of CZK 31851) spend 20.6% [16].

The following formula was used to calculate the amount available for food per day:

1 $$ \operatorname{Max}.\mathrm{cost}=\frac{\left(\mathrm{Parent}'\mathrm{s}\ \mathrm{income}\kern0.5em \mathrm{x}\ 2\right)+\left(\mathrm{child}\ \mathrm{benefit}\ \mathrm{x}\ 2\right)\ \mathrm{x}\ \mathrm{months}\ \mathrm{per}\ \mathrm{year}\ \mathrm{x}\ \left(\%\mathrm{of}\ \mathrm{income}\ \mathrm{spent}\ \mathrm{on}\ \mathrm{food}\right)}{\mathrm{Days}\ \mathrm{per}\ \mathrm{year}}, $$

Hence, the low income food budget was CZK 177.7 (€ 6.9) per day; and CZK 301.5 (€ 11.7) per day for a household on median income.

### Foods, their prices and their categorisation

In total, 330 foods and more than 3900 online prices were collected from three different food retailers’ websites in the Czech Republic (Nakup.itesco.cz, Rohlik.cz, Košík.cz) during May and June 2018. Data (name, brand, weight and price) were collected for raw/uncooked foods and ready-to-eat products. For all items the price per kilogram or litre was calculated. If prices were provided per piece (e.g. for avocados, melons, cauliflower), their reference weights were used to calculate the price per kilogram [21].

The foods, using their generic names, along with their median prices were organized into the same categories as defined in the National Food Consumption Survey 2016 conducted by the Czech Statistical Office [22]. The 11 categories include: Cereals, Meat and meat products, Fish, Milk and milk products, Eggs, Fats and oils, Vegetables, Pulses, Potatoes, Fruits and juices, and “Other foods” (such as condiments, sugar, sweets and confectionery). These foods were further organized into 79 sub-groups or individual foods [22]. An overview over the applied food categories and food groups along with examples and the consumed amounts is provided in Additional file 1: Table S1. The values reported in the Consumption Survey, which are stated as annual per capita averages in kilograms, were individually standardized for each family member proportionally to the estimated energy requirement per day (see below). Items from groups: tea and coffee; mineral waters; alcoholic and non-alcoholic beverages were excluded, based on the fact that these items are not recommended within FBDGs. Hence, the foods included in the food baskets without the food groups mentioned above cover all recommended nutrient intakes.

### Nutritional composition

The nutritional composition (43 nutrients) was collected from various databases. If unavailable from the Czech food composition database [23], the nutritional composition was obtained, in order of priority, from: the German Federal Food Code [24]; McCance and Widdowson’s ‘Composition of foods integrated dataset’ on the nutrient content of the UK food supply [25]; Fødevaredatabanken – Danish food composition database [26]; and The United States Department of Agriculture (USDA) National nutrient database for standard reference [27]. If foods were not consumed raw, preparation methods such as boiling, simmering or baking were considered during the calculation of the nutrient content. In addition to the values of the nutrients, the contents of fibre and water (for both raw and prepared foods), along with the value of the edible proportion, were recorded. The available nutrients from each food were calculated considering the corresponding yield factors, which depend on weight changes during preparation caused by heating and unavoidable food waste (such as pits, skin, bones) as described previously [28].

### Recommended energy and nutrient intakes

The German-Austrian-Swiss (DACH) population reference intakes, adopted and revised by the Czech Nutrition Society: Recommended Nutrient Intakes (RNIs), Estimated Energy Requirements (EERs) and Acceptable Macronutrient Distribution Ranges (AMDRs), were used as the reference for a nutritionally adequate diet [29]. For the RNI values not available for the Czech Republic, values recommended by the World Health Organization were applied [30, 31]. Macronutrient EERs and AMDRs are WHO recommendations, except omega-3 and -6 fatty acids, where combination of both, minimum from Czech and maximum from WHO, were applied.

### Linear programming

Linear programming (LP) is a mathematical method for the optimisation (=minimization or maximization) of a given linear goal function (= objective function), which is a loss function or its negative of the goal variable. This goal function is subjected to a set of constraints and is capable of changing list of decision variables until the constraints are met [32]. Here, the decision variables are the amounts of foods to be included in the optimized FBs. If a solution exists that is capable of meeting all constraints, this solution is called “feasible”; otherwise, the linear programming algorithm provides a non-feasible solution that approximates the set constraints as closely as possible. Constraints can be applied to the model by defining minimum or maximum thresholds for e.g. cost, nutrients, or the minimum or maximum weights of food groups recommended. In this study, only feasible solutions were accepted, meaning that all nutritional constraints were met (Table 1).

**Table 1:** Estimated energy requirements (EERs), acceptable macronutrient distribution ranges (AMDRs) and recommended nutrient intakes (RNIs) for each member of the reference family used as constraints during linear programming. The nutrient contents of all optimised food basket lie within the indicated ranges [29–31]

	Female (25–50 years)	Male (25–59 years)	Boy (7 years)	Girl (16 years)
Energy (kcal)	2617	3280	2025	2451
Protein (g)	65–90	58–111	26–69	58–94
Fat (g)	87–102	109–128	67.5–78.8	81.7–95.3
SFAs (g)	< 29.1	< 36.4	< 22.5	< 27.2
PUFAs (g)	17.4–29.1	21.9–36.4	13.5–22.5	16.3–27.2
n-3 PUFAs (g)	1.5–5.8	1.8–7.3	1.1–4.5	1.4–5.4
n-6 PUFAs (g)	7.3–23.3	9.1–29.2	5.6–18.0	6.8–21.8
TFAs (g)	< 2.9	< 3.6	< 2.3	< 2.7
Added sugar (g)^a^	< 32.7	< 41.0	< 25.3	< 30.6
Cholesterol (mg)	< 300	< 300	< 300	< 300
Carbohydrate available (g)	360–491	451–615	278–380	337–460
Fiber (g)	≥ 25.0	≥ 25.0	≥ 25.0	≥ 25.0
Na (mg)	< 2000	< 2000	< 2000	< 2000
K (mg)	≥ 2700	≥ 2700	≥ 2700	≥ 2700
Ca (mg)	≥ 1000	≥ 1000	≥ 900	≥ 1200
Mg (mg)	≥ 300	≥ 350	≥ 170	≥ 400
Fe (mg)	≥ 16	≥ 10	≥ 10	≥ 12
Zn (mg)	≥ 7.0	≥ 10.0	≥ 7.0	≥ 7.0
Se (μg)	≥ 70	≥ 30	≥ 20	≥ 70
Iodine (μg)	≥ 200	≥ 200	≥ 140	≥ 200
Vit A-RAE^b^ (μg)	≥ 800	≥ 800	≥ 800	≥ 900
Thiamine (mg)	≥ 1.0	≥ 1.2	≥ 1.0	≥ 1.0
Riboflavin (mg)	≥ 1.2	≥ 1.5	≥ 1.1	≥ 1.2
Vit B6 (mg)	≥ 1.2	≥ 1.5	≥ 700	≥ 1.2
Vit B12 (μg)	≥ 3.0	≥ 3.0	≥ 1.8	≥ 3.0
Vit C (mg)	≥ 100	≥ 100	≥ 80	≥ 100
Vit E (mg)	≥ 14	≥ 14	≥ 10	≥ 15
Folate (μg)	≥ 400	≥ 400	≥ 300	≥ 400
Niacin (mg)	≥ 13	≥ 16	≥ 12	≥ 13

^a^Added sugars were all mono-and disaccharides that were not part of fruits, vegetables, milk and milk products but originated from beet root, honey, maple syrup etc

^b^ *RAE* Retinol Activity Equivalents

In LP models, constraints that determine the extent to which the objective function can be minimized or maximized are called “active” constraints [33] and it has been observed that, in FBs, only a few nutrients that meet exactly 100% of their reference value are “active” constraints [34]. Linear optimization was done with the COIN-OR CBC optimization engine algorithm, which is part of the open-source add-in OpenSolver (v. 2.9.0) for MS Excel® [35]. LP was used to separately develop FBs for each member of the reference family and then combined for the whole family.

### Cultural acceptability of Czech food baskets

It has been understood a long time ago that when applying LP to generate FBs that are optimised for cost, the solution comprises just a few foods (typically below a dozen), which limits its practicability due to a lack of food diversity [28, 34, 36–38]. Recommendations on food intake are only followed when they are culturally acceptable, but cultural acceptability is a social construct hardly to be measured directly [39]. Therefore, we used the relative deviation (RD) of the optimised FBs from the reported food intake of Czechs, based on the foods and food groups used for the latest food consumption survey [22] as a proxy for cultural acceptability. For example, if the reported average intake of pasta by the reference family would be 50 g/day and the optimised FB would suggest 60 g/day, the RD for this food would be + 20% (Formula 2).

2 $$ {RD}_i=\frac{m_i-{M}_i}{M_i} $$

In Formula 2, *m* stands for the optimised weight of the *i*-th food or food group in grams in the food basket after optimization and *M*_*i*_ is the weight of the same food or food group reported to be consumed by the corresponding family member [22]. This calculation was done using the raw versions of the foods while the nutrient content was calculated from the prepared food variants. In order to ensure highest possible similarity of the optimized FB, we minimized the sum of all absolute (= non-negative) values of relative deviations of FBs for all foods/food groups, for which the abbreviation TRD (total relative deviation) has been used in the following (Formula 3). In Formula 3, N stands for the number of foods/food groups (here: 79).

3 $$ TRD={\sum}_{i=1}^N abs\left({\mathrm{RD}}_i\right) $$

Hence, the lower TRD, the more similar is the optimised FB is to the Czech dietary intake [22]. The value of TRD varies depending on the number of food groups, which makes a comparison with other similar investigations difficult. Therefore, we used the average relative deviation (ARD) of the FB from the food intake data as a proxy for the relative similarity or difference between the reported food intake and the optimized FBs. The ARD was calculated by dividing the TRD by the number (N) of food groups (Formula 4).

4 $$ ARD= TRD/n $$

The ARD therefore indicates the average deviation of the calculated FB from the observed consumption patterns of the individual relative deviations of all family members’ food baskets combined.

In this study four different models, or family FBs, were constructed to investigate the intercorrelations between cost and nutritional adequacy: Lowest cost FB (LCFB); *Food Basket of a Family with an Unconstrained Food Budget* (UCFB); *Food Baskets Optimized for Cultural Acceptability in a Low-Income Family on Minimum Wage* (MWFB); and *Food Basket for a Family on Median Income* (MIFB). Table 2 gives an overview over which goal functions and which sets of constraints were applied to the individual models. For all FBs, the (decision) variables calculated were the amounts and numbers of foods selected in order to fulfil a household’s enforced recommendations per day or month. The outputs include the composition of the food baskets, their cost, and their average relative deviation (ARD) from the food consumption patterns in the Czech Republic [22]. To allow a sufficiently diverse representation of mid-sized and large food groups, the enforcement of a minimum number of food items per group was investigated: groups of up to five foods comprised at least one food item; groups having more than 5 but less than 10 items had at least two foods; large food groups (e.g. fish, nuts, cheese), comprising at least 10 foods had at least three food items in the diversified versions of the FBs [34].

**Table 2:** Names, objective functions, and sets of constraints applied to each of the four food baskets calculated

Model names and acronyms	Objective function	Set of constraints enforced
Lowest-Cost Food Basket (LCFB)	Cost (min)	EERs, AMDRs, RNIs
Budget-Unconstrained Food Basket Optimized for Cultural Acceptability (UCFB)	TRD (min)	EERs, AMDRs, RNIs
Food Basket for a Family on Median Income Optimized for Cultural Acceptability (MIFB)	TRD (min)	EERs, AMDRs, RNIs, MIFC
Food Baskets Optimized for Cultural Acceptability in a Low-Income Family (MWFB)	TRD (min)	EERs, AMDRs, RNIs, MWFC

*TRD* total relative deviation, *EERs* estimated energy requirements, *AMDRs* acceptable macronutrient distribution ranges, *RNIs* recommended nutrient intakes, *MIFC* median income food budget (CZK 301.5), *MWFC* minimum wage food budget (CZK 177.7)

As the definition of a diet implicates not only the food and drink provided by the optimised food baskets but also habitual nourishment, the current study did not aim at defining optimised diets.

### The lowest-cost food basket (LCFB)

The LCFB was calculated to investigate the absolute lowest cost of a food basket that fulfils all Czech and WHO nutrient recommendations, including EERs, AMDRs, and RNIs [8, 29, 40]. The LCFB was optimised using the minimised cost as goal function and does not consider dietary diversity or cultural acceptability (Table 2).

### Budget-unconstrained food basket optimized for cultural acceptability (UCFB)

AS for the LCFB, the UCFB meets all Czech [29] and WHO nutrient recommendations [8, 40]. The goal function applied was the minimum of the total relative deviation (TRD). The food profile of the UCFB matches the reported food intake by Czechs the best, without considering any cost constraints. In order to achieve realistic and practical portion sizes the calculated daily amounts were multiplied by 30.4 (=average number of days per month) in order to create average monthly food baskets for one household. The share of the budget for each family member for the UCFB (and MIFB + MWFB) was calculated based on cost proportions found in the lowest-cost food basket (LCFB).

### Food basket for a family on median Income optimized for cultural acceptability (MIFB)

The reference Czech family of four living on median salaries has a daily food budget of CZK 301.5 (€ 11.7 EUR). The MIFB was calculated to meet all the nutrient recommendations (EERs, AMDRs, RNIs) within the median wage cost constraint (CZK 301.5) and, in addition, the goal function aimed to calculate the least possible total relative deviation (TRD) from the Czech food intake survey [22].

### Food baskets optimized for cultural acceptability in a low-income family (MWFB)

The MWFB was calculated similar to the MIFB, by enforcing the nutrient recommendations (EERs, AMDRs, RNIs) and within the minimum wage cost constraint of CZK 177.7 (€ 6.9), but in addition, as a goal function, aimed to calculate the least possible TRD from the Czech food intake results [22].

## Results

### Nutrient deficiencies at given energy provision

As reported by the national Czech food consumption survey [22], the Czech reference family consumed too little vitamin D, folic acid and calcium (17, 39 and 76% of the RNI, respectively) and too large amounts of added sugar and sodium (2.2-fold and 2.9-fold value of the recommended upper intake). The reported daily food intake was represented by 159 foods and costed CZK 592.25 (€ 22.98).

### The Czech food basket with the lowest cost (LCFB)

In order to identify the composition of a Food Basket which fulfils all nutritional requirements for a Czech family of four at lowest possible cost, the food supply was optimized for cost, applying nutrient constraints only. This LCFB contained 13 different foods and costed CZK 128.2 (€ 4.95) per day (Table 3). The average relative deviation (ARD) of this LCFB per food group was 686% from Czech food intake patterns.

**Table 3:** Composition of The Czech food basket with the highest affordability (LCFB)

Category	Food (group) name, English	RD	Weight, raw (g)	Cost (CZK)
Milk	Milk, skimmed	+ 473%	946	13.2
Vegetables	Iceberg Lettuce	+ 181,560%	9947	32.7
Cereals	Wheat flour, medium-ground	+ 391%	1058	15.2
	Wheat flour wholegrain		243	8.5
	Barley groats	+ 1783%	98	2.5
	Pasta, (without egg)	+ 1915%	392	13.2
Nuts & seeds	Poppy seeds	+ 2777%	32.6	3.8
Meat	Liver, chicken	−56%	11.3	2.1
Fish	Herring in oil	−19%	109	1.9
Fats & oils	Pork lard	+ 786%	183	8.9
	Vegetable fat spread(72% fat)	+ 345%	26.3	18.3
	Olive oil		33.9	7.6
	Salt	−41%	9.3	0.3
		Sums	13,089	128.18

*RD* relative deviation

The nutrients that work as active constraints and determine the overall cost of FB are stated in Table 4. The cost determining nutrients depended on the sex and age of the individuals members of the family. Lowering the lower limits and increasing the upper limits of these nutrients for each family member would result in reduced cost of the food basket. Nutrients not listed in Table 4 are automatically covered by the LCFB without causing any additional cost.

**Table 4:** The cost-determining nutrients in the LCFB. “AC” indicates the corresponding nutrient to be an active constraint that determines the overall cost of the LCFB

	Lower limits				Upper limits			
	Mother	Father	Girl	Boy	Mother	Father	Girl	Boy
Protein (g)					100%			100%
Fat (g)	AC	AC	AC	AC				
SFA (g)					AC	AC	AC	AC
n-3 PUFA (g)	AC	AC	AC					
n-6 PUFA (g)					AC	AC	AC	AC
Added sugars								AC
Riboflavin (μg)				AC				
Niacin (μg)	AC	AC		AC				
Folic Acid (μg)	AC	AC	AC	AC				
Vitamin C (mg)	AC		AC	AC				
Vitamin E (μg)				AC				
Calcium (mg)	AC	AC	AC	AC				
Potassium (mg)				AC				
Sodium (mg)					AC			AC
Iodine (μg)	AC	AC	AC					
Selenium (μg)	AC		AC					

### Changes of food diversity and similarity to the reported food intake depending on cost

**Fig. 1:**
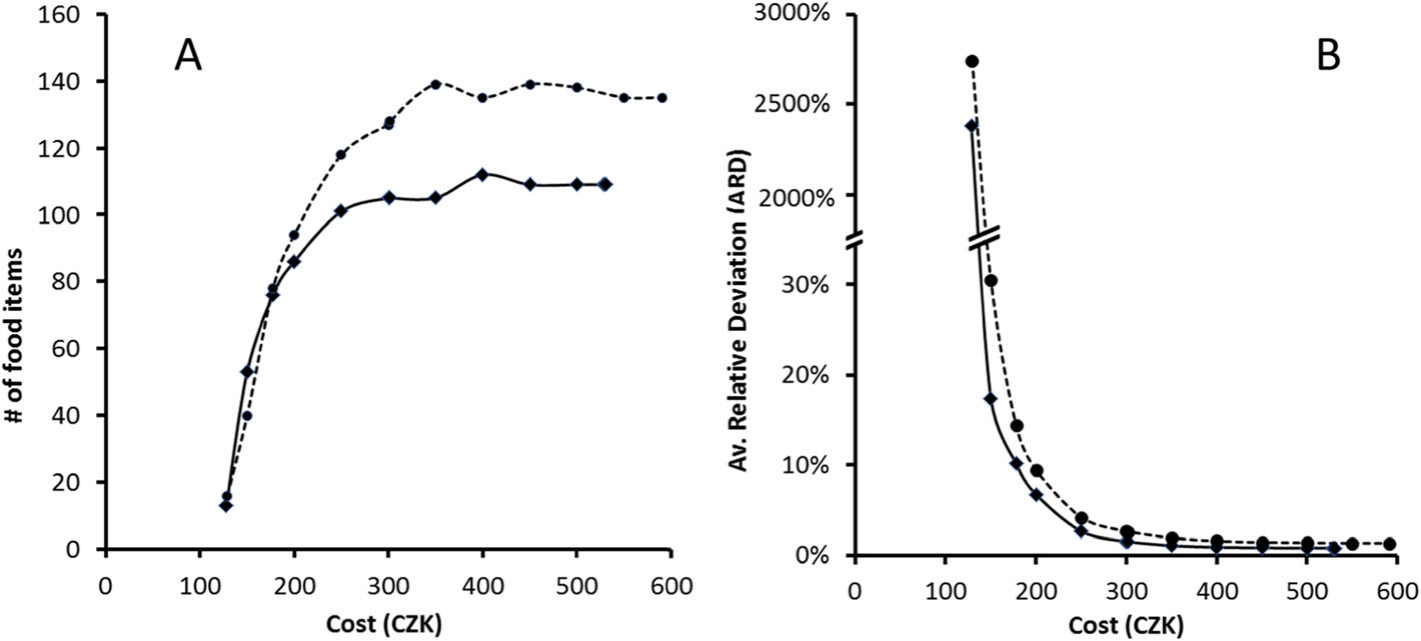
The effect of cost constraint on the food diversity (number of food items) in the FB for a Czech family of four (Panel A) and on the similarity to the reported intake of 79 food groups, expressed as the average relative deviation (ARD) (Panel B). The solid line refers to the non-diversified versions of the FBs while the dashed line indicates the versions of FBs where mid-sized food groups (6–9 items) contain at least two foods and large groups (10 or more items) comprise at least three foods

Up to an overall cost of CZK 300–350, the number of food items in the FBs for the Czech reference family increased at higher cost and remained unchanged beyond this cost level (Fig. 1, Panel A). Already a minor increase in cost beyond that of the LCFB resulted in a considerable gain in similarity to the reported intake of 79 food groups of the Czech family. While the least-cost versions of the FBs (LCFB) showed an extreme deviation from the reported food intake with and ARD of 2381 and 2744% for the non-diversified and diversified versions, this value dropped below 10% in a cost range between CZK 180 and 200 (Panel B). Beyond this cost level, additional constraints on the minimum number of foods can be implemented into LP to achieve a higher number of foods, and hereby higher food diversity, without causing higher cost.

### Food baskets optimized for cultural acceptability in a low-income family on minimum wage (MWFB)

The total daily food budget of the low income family was CZK 177.7 (€ 6.9) in 2018. The MWFB was optimized for cultural acceptability by reducing the deviation from the food intake data and the resulting basket. The MWFB consists of 74 foods, occurring in 65 groups, with an average relative deviation of 10.2% from the reported food intake patterns [22]. The father’s basket was the most expensive with a cost of CZK 55.7 (€ 2.1) per day, followed by the mother’s with a cost of CZK 42.8 (€ 1.7) per day. The basket for the girl is CZK 41.8 (€ 1.6) per day, followed by the boy’s costing CZK 37.4 (€ 1.5) per day.

The 74 food items (in total, 5994 g of food per day) were merged into 13 food groups depending on the foods’ similarity and based on the data from the National Food Consumption Survey 2016 [22]. The food groups along with their corresponding weights and cost are shown in Table 5.

**Table 5:** Amount of food in the categories and their cost in the food basket for a family on minimum wage (MWFB)

Food category	Weight (raw, g)	Weight share	RD	Cost (CZK)	Cost share
Milk	2214	36.9%	+ 483%	30.8	17.3%
Milk products	161	2.7%	−50%	10.1	5.7%
Vegetables	547	9.7%	+ 6%	18.4	10.3%
Pulses	18	0.3%	< 1%	1.1	0.6%
Potatoes	434	6.7%	−8%	8.7	4.9%
Fruits & juices	250	4.2%	−74%	11.8	6.7%
Nuts	25	0.4%	<+ 1%	3.4	2.1%
Cereals	1686	28.1%	+ 20%	35.2	19.8%
Meat & offal	241	4.0%	−52%	19.7	11.1%
Fish	27	0.4%	−17%	4.5	2.6%
Fats & oils	288	4.8%	+ 69%	27	15.2%
Other	90	1.5%	−73%	6.4	3.4%
Salt	12	0.2%	−66%	0.3	0.2%
Sums	5994	100%		177.7	100%

*RD* relative deviation from the reported intake of the corresponding category (=100%)

In order to reach nutritionally adequate and culturally acceptable baskets within a budget of CZK 178 per day for a family of four, the MWFB model suggests a considerably higher intake of skimmed milk, namely 1.8 L per day more than the family usually consumes. Moreover, the MWFB suggests an increased consumption of “Fats and oils” and a moderately elevated intake of Cereals and Vegetables (Table 5). In addition, the MWFB suggests that salt, meat, milk products as well as “Fruits and fruit juices” should be decreased by at least half of the amount reported to be consumed. For the “Other” category, which includes sugar and confectionary, the model suggest this be reduced by about 73% (− 159 g/day). In the MWFB, large groups such as Vegetables, Cereals and “Fruits and juices” are represented by a large variety: the Vegetable category comprises 19 different items with a strong focus on roots and tubers, bell pepper, onions, carrots, and cole crops such as cabbage, cauliflower and broccoli. Likewise, the Cereals group comprises a variety of grains and “Fruits and juices” are represented by 12 different foods items, though juices are missing in the latter category (Additional file 1: Table S2).

The most expensive category in the MWFB is Cereals, followed by milk and meat, the latter two summing up to about half of the overall cost of the MWFB (Table 5).

### Food basket for a family on median Income (MIFB)

The MIFB contains 105 foods grouped into 79 different subcategories, which makes 31 more foods compared with the LCFB. The higher family budget, and relaxed cost constraint, resulted in a low average relative deviation (ARD) of only 1.5% from the food intake data. The proportion of budget share in the MIFB remained the same as in the LCFB. The 105 foods and 79 subcategories were merged into 14 food groups (Table 6), depending on the foods’ similarity and based on the categorisation applied in the National Food Consumption Survey 2016 [22]. In contrast to the LCFB, eggs were included in the MIFB.

**Table 6:** Amount of food in the categories and their cost in the food basket for a family with a median income (MIFB)

Food category	Weight (raw, g)	Weight share	RD	Cost (CZK)	Cost share
Milk	1042	17.4%	+ 175%	14.5	%
Milk products	345	5.8%	+ 7%	28.9	9.6%
Eggs	87	1.5%	0%	8.8	2.9%
Vegetables	547	9.1%	0%	22.0	7.3%
Pulses	18	0.3%	0%	1.2	0.4%
Potatoes	434	7.3%	0%	10.0	3.3%
Fruits & juices	972	16.2%	0%	47.6	15.8%
Nuts	23	0.4%	−11%	3.6	1.2%
Cereals	1688	28.2%	+ 20%	73.8	24.5%
Meat	379	6.3%	−25%	42.0	13.9%
Fish	32	0.5%	0%	4.9	1.6%
Fats & oils	205	3.4%	+ 20%	22.1	7.3%
Other	208	3.5%	−38%	21.2	7.0%
Salt	6	0.1%	−83%	0.2	0.1%
Sums	5986	100%		301.5	100%

*RD* relative deviation from the reported intake of the corresponding category

In order to reach nutritionally adequate and culturally acceptable baskets within a budget of CZK 301.5 (11.7 EUR) per day for a family of four, the MIFB model suggests still a considerable increase in whole milk of 0.7 L per day, but the suggested increases for the other categories are quite moderate: + 20% Cereals and “Fats and oils”. In addition the MIFB suggests that: the daily intake of salt to be decreased by 83% (− 29.7 g); meat to be decreased by 25% (− 125 g) (Table 6). There is no suggestion to deviate noteworthy in the categories: Fish, Nuts and seeds, Potatoes, Pulses, Vegetables and Eggs in the MIFB compared with the reported food intake data.

The most expensive category in the MIFB is Cereals, followed by Meat and “Fruits and juices”. These three categories sum up to more than half of the overall cost. Milk products, Vegetables, “Fats and oils” and “Other” cause about one third of the overall cost with comparable shares (7–10%) (Table 6).

### Budget-unconstrained food basket (UCFB)

Beyond an overall cost of CZK 528.4 (€ 20.6) per day, the composition of the FB, which is both nutritionally adequate and maximally similar to the observed Czech food intake patterns [22], does not change anymore. The suggested changes occurring in this UCFB are exclusively based on nutritional constraints as the cost constraint stops to be “active”. The UCFB consists of 110 foods and deviates from food intake data on average by 0.8%. The maximum relative deviation is − 85% and relates to salt, which results in 30.4 g reduction compared with intake data. The UCFB model suggests only a 45% increase (+ 169 g per day) in the amount of skimmed milk.

**Fig. 2:**
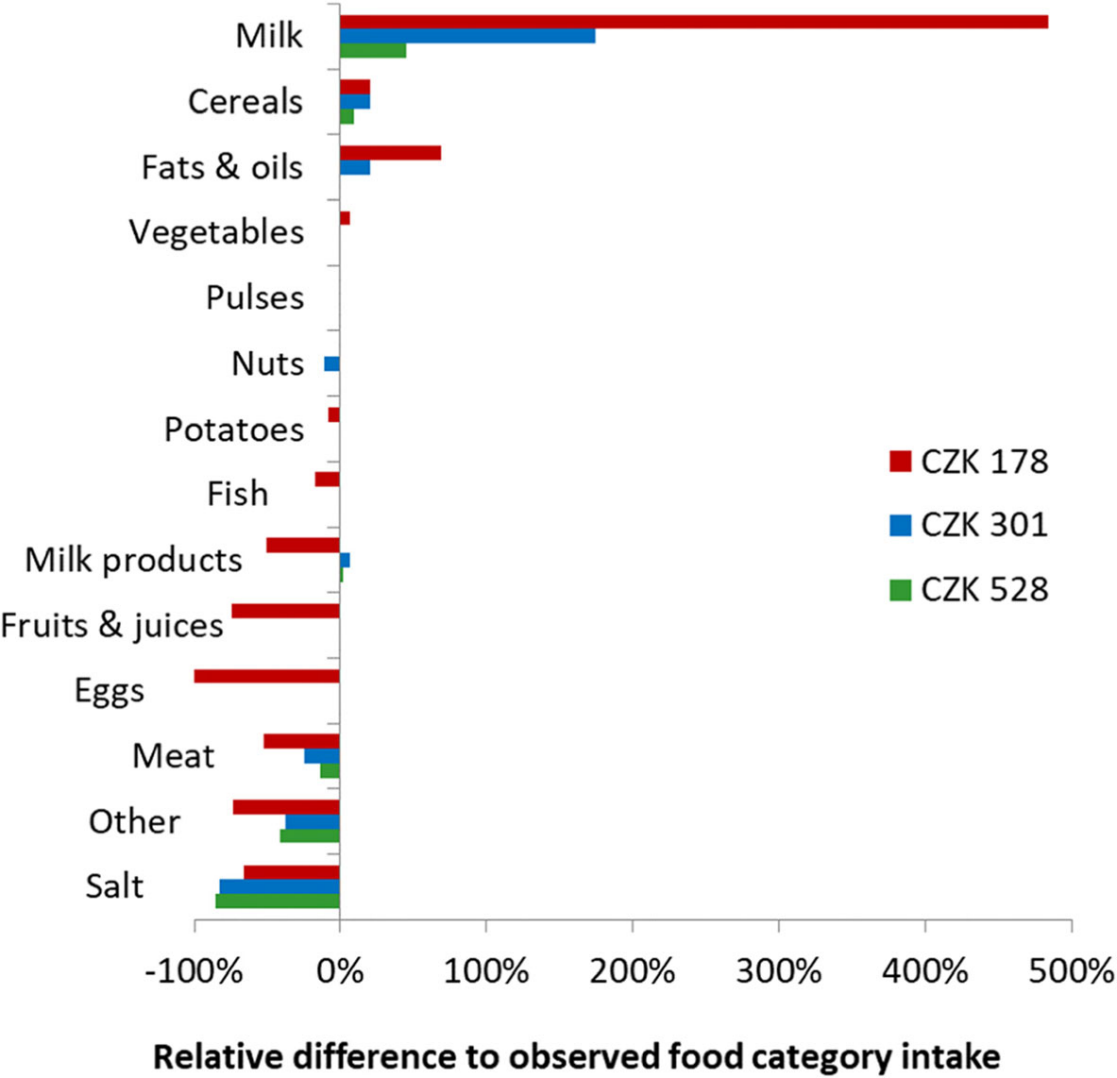
Relative dietary adjustments of the three calculated Food Baskets. Percentages indicate the relative difference of the optimized FBs to observed food group intake

A comparative overview of the relative deviations of the MWFB, the MIFB and the UCFB from the food intake data is provided by Fig. 2. The deviations are the highest in the MWFB, for families living on the minimum wage, followed by the MIFB and then the UCFB. In the latter, the deviations are based on prevailing nutritional inadequacy of the reported dietary intake only.

For some categories, the changes suggested by all three models go in parallel. A reduction of salt; Meat; items of the “Other” category and a higher intake of Cereals are suggested by all three optimisations. Other categories are either not noteworthy affected in any of the models (Pulses, Potatoes and Nuts) or their suggested intake depends on the available budget, i.e. that of Milk and “Fats and oils”; the suggested consumption of these two commodities increases under cost pressure. The recommended amounts of Eggs, “Fruits and juices”, Milk products, and Fish decrease under cost pressure (Fig. 2). Reductions in the “Other” category primarily refer to reduced amounts of sugar, chocolate, cocoa products, sweets, preserves and similar.

## Discussion

The optimization of a food basket for a Czech family of four has shown that it is possible for the family to obtain a nutritionally adequate, yet low-cost diet. The budget of CZK 128 (€ 4.9) was identified as minimum to afford a diet fulfilling the EERs, AMDRs, and RDIs for this family. The cost of CZK 128 (€ 4.9) is slightly higher compared to what researchers found in other European countries such as Denmark, where the cheapest FB for a family of four cost approximately DKK 27 (€ 3.6) per day [28]. The most affordable food basket contains only 13 food items, which in addition to creating a very monotonous diet is a diet very low in dietary diversity and so should not be recommended as a basis for FBDG in Czech Republic. Besides the poor food diversity, the suggested daily amounts of single foods for the reference family, i.e. almost 10 kg of eisberg lettuce, are inappropriate for human consumption and therefore culturally unacceptable. In order to increase the attractiveness and social acceptability of the food basket composition and more realistically align with Czech consumer’s expectations, further calculations were necessary to minimize the deviation from current dietary patterns.

In the LCFB, food items with good nutrition-to-cost ratio were selected, suiting the approach of „nutrient profiling“. Nutrient profiling ranks food items based on their nutrient content and can help to identify foods with high nutritional quality for their price, including their contribution to a healthy diet [41]. Food items such as milk, lettuce, wholegrain flour, barley groats, pasta, chicken liver, herring, vegetable fat spreads, olive oil and poppy seeds were selected by the algorithm in the lowest-cost FB. These foods are part of food groups including: fruit, vegetable, whole grains, refined grains, milk, (vegetable) fats, and nuts & seeds which have a good nutritional quality relative to their price [41]. The nutrients listed in Table 4 were identified as price-determining and are the ones that consumers actually pay for, other nutrients are naturally present in sufficient amounts. For example, if the RNI for calcium will decrease for each family member or more low-cost foods with sufficiently high calcium content will be available, the FB will become cheaper. The same would apply if there was no upper RNI for sodium for mother and boy. Nevertheless, one of the main limitations of the LCFB, along with its low dietary diversity, is its high deviation (686% on average) from the reported food intake patterns of the Czech population.

In order to address this, the relative weight difference of 79 food groups to the food intake data was minimised, while the overall maximum cost of foods and nutritional adequacy were all enforced by LP, in order to design a FB that fulfills the EERs, AMDRs and RNIs, is culturally acceptable and affordable. This resulted in an optimised minimum wage family food basket (MWFB) costing CZK 178 (€ 6.9) with an average deviation of 10.1%. This can be considered as being fairly similar to reported food intake patterns while offering an opportunity for change towards healthier diets. For example, this food basket is based on a 66% reduction of salt content compared with food intake data along with an inreased consumption of vegetables and cereals, the major part of the latter being wholegrain (Fig. 2).

According to the Ministry of Health of the Czech Republic, the average national daily salt consumption in 2016 has been about 15–16 g and hereby clearly above the recommended limits of 5 g per day [42]. This intake was also higher than averge daily European consumption of 8–12 g. High salt intake contributes to high blood pressure and increased risk of cardiovascular disease [43], which are the most prevalent causes of death in the Czech Republic [16]. Reduction in salt intake was identified as one of the most cost-effective measures to improve population health, especially in terms of preventing CVDs [43] and this is recognized by the Czech Ministry of Health. The nutritional composition of the MWFB additionally supports a reduction in CVD and high blood pressure incidence by guaranteeing a sufficiently high intake of potassium (Table 1), which is considered to counteract the adverse health effect of elevated sodium intake [43].

Although the food baskets were optimized on nutrient-based constraints, epidemiological studies rather focus on the health effects of foods and food groups. The MWFB suggests decreased consumption of meat by 52% compared with current consumption trends of the Czechs. The average daily intake of meat and meat products by Czechs was in 2016 about 220 g per day. There is convincing evidence that an elevetad meat intake is associated with increased risk of developing cancer, primarily in the colorectum [44] and the suggested reduction of meat intake can be assumed to contribute to a reduction of this risk. This risk reduction may further be supported by the significantly higher amounts of milk as a higher intake of calcium-rich diets is associated with a lower incidence of breast and colon cancer [44]. This effect, however may be limited in the MWFB as, although the amount of milk is considerably increased, the content of milk products is moderately decreased.

The MWFB, if used as a basis for FBDG would also help to promote increased consumption of vegetables – only 9.1% consume the recommeded intake of 5 or more portions per day and more than 43% of Czechs reported not to achieve at least one portion per day [7]. The amount of vegetable in this MWFB is suggested to increase by 6%, while fruits and juices should be reduced by 74%. The nutrients found in fruits and juices were calculated to be more expensive than those in vegetables. In the case of imported fruits, transport and storage costs determine fruit to be „luxury goods “for Czech low-income families and juices were not included at all in the MWFB (Table S2). Consumption of cereals is suggested by the MCFB model to increase by 20% and due to the lower cost includes mostly unprocessed items, such as wheat and rye flour, being in part as wholegrain variants.

The amount of milk in the FB is suggested to be increased by 484%, while, at the same time, milk products should be reduced by 50%. This recommendation is based on the fact that the nutrients present in milk become more expensive when processed into milk products. The suggested reduction of sweets and confectionery from category „Others “complies with the WHO recommendation of decreasing sugar intake to less than 5% of the total energy intake [45] in order to maintain a healthy body weight and prevent dental caries. High intake of low-energy-density foods such as vegetable and cereals has been associated with lower body weights and overall better health [9].

The aim of this study is to recommend how to modify the Czech Food Based Dietary Guidelines when a cost constraint is considered for low income consumers. Therefore the recommended nutrient intakes (RNIs), within pre-defined energy amounts, were applied to ensure all nutrients are covered by the foods in the food baskets. The results support the development of Czech FBDG in terms of proportion of the food groups that should be recommended overall, including specific food items. Moreover, the financial affordability of a nutritionally adequate diet, where only the cost of the raw foods are considered, has been calculated. Also in what proportion each food group comprise the total cost is calculated, which may be useful for Czech authorities either to better plan social benefits or other measures to help low income families out of poverty. In addition such proportions may help guide agriculture or horticulture policies in Czech and national food security measures regarding what foods to produce and which to import. Several non-EU governments, such as Canada, Australia and the US, use LP for estimation of how much money their population need to afford nutritionally adequate diet [28]. This study could therefore support national government in planning social and welfare policies.

Based on the comparison of the generated MWFB and reported food intake patterns of the Czech population in 2016, the FBDGs for a low-income Czech family of four could include the considerations listed in Table 7.

**Table 7:** Recommendations for a Czech family of four living on minimum wage to achieve a food supply that is nutritionally adequate and that is as similar as possible to observed food supply patterns in the Czech Republic

- Increase your vegetable consumption to up to 570 g per day	
- Consume by 20% more cereals, preferably wholegrain	
- Drink more milk every day, up to 2.2 l daily	
- Reduce consumption of milk products, such as cheese or yoghurts by half	
- Consume more vegetable-based fats and oils up to 290 g per day	
- Reduce your meat consumption by half, to about 200 g per day	
- Reduce consumption of fruits and juices by 2/3 to about 190 g per day	
- Eat by 60% less salt and no more than 12 g per day	
- Reduce consumption of sweets and confectionery, especially sugar and biscuits to 1/3	
- Consume on average around 20 g of nuts and seeds, such as peanuts or sesame seeds per day	
- Consume on average around 40 g of pulses, such as yellow peas or beans per day (~ 20 g raw weight)	

Vitamin D has not been considered in the list of minimum thresholds during the calculations, as it’s not part of the micronutrients for which a RNI value has been released for the Czech Republic [29]. A recent study by *Bischofova* et al. state that dietary intake of vitamin D was in more than 95% of Czech population below the recommended RDIs [46]. Public health interventions directly promoting intake of vitamin D, through supplements, could be more effective. Evaluation of this intervention would be required and compared with promotion of a higher vitamin D intake through FBDGs. The main dietary source of vitamin D among Czechs was eggs [46], and this food group was absent from the MWFB for low-income family. Preliminary calculations identified fatty fish to be the most cost-effective source of vitamin D, but the cultural acceptability among the Czech population and its local cost would need to be overcome.

Even though *Maillot M* et al. argue that foods with the least favourable nutrient profile, for example sweets or salty snacks, can still contribute up to 41% of the energy intake to a nutritionally adequate dietary pattern [47], items such sweetened beverages and sweets were not included in the LCFB by the LP algorithm. However, optimizing for similarity, a certain amount of foods from this category (and even alcoholic beverages) would have been enforced into the FB for low-income and median-income families. Given that usual dietary habits play a major role in preferred dietary choices [48], the elimination of these “unfavourable” foods might increase the reluctance of low-income consumers to adopting FBDGs. However, foods or beverages considered to have the potential to harm human health such as alcohol should not become part of the recommendations.

### Limitations

The cost of the food baskets in this study only applies to the purchase of food and it does not include expenses associated with food storage (cooling, freezing) and food preparation such as those linked to energy, transport, cooking equipment and time. The FBs are designed for a reference Czech family of four and do not apply to people who are outside the reference age ranges and to individuals with special nutritional needs such as pregnant women and people with food intolerance or allergy. Cost linked to avoidable food waste or foods that spoil after purchase are not considered. The prices of foods used in this study were collected online and may moderately differ from prices effective in supermarkets and at other retailers. However, as the online trading of foods has to stay competitive to the over-the-counter selling, major differences in food prices are unlikely.

## Conclusion

The main goal of this study was to develop food baskets for a Czech family of four with low socio-economic status, living on minimum monthly wages. It has been demonstrated that nutritionally adequate foods, but not diets, are affordable for families living on the minimum wages. The results of this study present foods and proportions of food categories which are necessary for a nutritionally balanced diet (Table 7). The study suggests the food basket MWFB is the FB that is recommended to form the basis of Czech FBDGs for low income families. Implementation of these FBDGs would assist in the prevention of both micronutrient deficiencies and NCDs such as high blood pressure, type 2 diabetes, CVDs and obesity.

Preparation of meals by low income families based on these FB could be a topic for further investigations including qualitative studies on acceptability of the suggested food supply. Further research investigating other barriers towards compliance with FBDG among Czech consumer would allow more targeted implementation and promotion of guidelines.

This study can promote constructive discussion among nutritionists and policy makers in the Czech Republic. Inter-sectoral collaboration among various ministries, agriculture, horticulture, health and education, is required where they the implications of this study can be discussed with regard to helping to reduce social inequalities in Czech society.

## Supplementary information

**Additional file 1: Table S1.** List of foods and food groups along with examples and their numbers included into the optimization. **Table S2.** Amounts of single foods and their cost in the food basket for a family on minimum wage (MWFB) on a daily and monthly basis. RD, relative deviation from the reported intake of the corresponding category.

## Data Availability

The single food prices and the extracted food composition data used in this study are available from the corresponding author on reasonable request.

## Abbreviations

AC: Active constraint
AMDRs: Acceptable macronutrient distribution range
ARD: Average relative deviation
CVD: Cardio-vascular disease
EER: Estimated energy requirement
FB: Food basket
FBDGs: Food-based dietary guidelines
LCFB: Lowest-cost food basket
LP: Linear programming
MIFB: Food Basket for a family on median income
MWFB: Food baskets for a low-income family
RD: Relative deviation
RNI: Recommended nutrient intake
TRD: Total relative deviation (=sum of RDs of all food categories)
UCFB: Budget-unconstrained food basket
