# Supplementary material for: Nutritionally adequate food baskets optimised for cultural acceptability as basis for dietary guidelines for low-income Czech families
Source: Nutr J. 2019 Dec 6;18:84. doi: 10.1186/s12937-019-0510-y (PMC6898948; doi:10.1186/s12937-019-0510-y)
Supplement: Supplementary file 1 — Additional file 1: Table S1. List of foods and food groups along with examples and their numbers included into the optimization. Table S2. Amounts of single foods and their cost in the food basket for a family on minimum wage (MWFB) on a daily and monthly basis. RD, relative deviation from the reported intake of the corresponding category. [file 12937_2019_510_MOESM1_ESM.docx]

Table S1. List of foods and food groups along with examples and their numbers included into the optimization

| **Food group** | **Subgroup** | **Examples** | **# of foods** |
| --- | --- | --- | --- |
| Cereals | Breakfast cereals | Corn flakes | 1 |
|  | Rice | Rice (basmati, brown) | 2 |
|  | Cereals, other | Couscous, quinoa, buckwheat, muesli, puffed rice | 7 |
|  | Wheat flour | Wheat flour | 3 |
|  | Rye flour | Rye flour | 1 |
|  | Barley and oat products | Barley & oat flakes | 3 |
|  | Spelt products | Spelt flour | 3 |
|  | White bread | Toast bread, wheat, baguette, cereal buns, white bread | 9 |
|  | White bread, varieties | Wheat roll, pita | 2 |
|  | Bakery products and dumplings | Sponge cake, croissant, Christmas cake, dumplings, pizza | 9 |
|  | Pasta | Pasta and noodles (with egg) | 4 |
| Milk | Drinking milk, cow | Milk, whole, semi-skimmed, skimmed, flavoured | 5 |
|  | Drinking milk, goat | Goat milk | 1 |
| Milk products | Cheese | Cheddar, gouda, brie, feta, camembert, mozzarella | 23 |
|  | Curd and cottage cheese | Cottage cheese, curd | 2 |
|  | Other milk-based products | Yoghurt (natural & flavoured), kefir, skyr, cream, buttermilk | 16 |
| Eggs | Eggs | Hen eggs | 1 |
| Vegetables | Cucumber | Cucumber | 1 |
|  | Tomatoes | Tomatoes, fresh and dried | 5 |
|  | Bell pepper | Bell pepper (red, green, yellow) | 3 |
|  | Gherkins | Gherkins | 1 |
|  | Cabbage | White cabbage (fresh & canned) | 4 |
|  | Kale, Brussels sprouts | Kale, Brussels sprouts | 2 |
|  | Cauliflower | Cauliflower | 1 |
|  | Kohlrabi, radish | Kohlrabi, radish | 3 |
|  | Onions | Onions (red & white), spring onion | 3 |
|  | Garlic | Garlic | 1 |
|  | Lettuce, iceberg, ruccola | Lettuce, iceberg, ruccola | 4 |
|  | Spinach | Spinach (fresh & frozen) | 4 |
|  | Carrot | Carrot | 2 |
|  | Parsley, tuber | Parsley, tuber | 1 |
|  | Celeriac, tuber | Celeriac, tuber | 1 |
|  | Melons and pumpkins | Melons: cantaloupe, water, butter squash, Hokkaido | 5 |
|  | Green peas | Green peas | 3 |
|  | Green beans | Green beans | 2 |
|  | Mushrooms | Mushrooms | 4 |
|  | Vegetables, other | Broccoli, beetroot, fennel, ginger, chicory, leak, eggplant | 16 |
| Pulses | Beans | Beans (dried and baked, canned) | 2 |
|  | Peas | Peas (yellow and green), chickpeas, tofu | 6 |
|  | Lentils | Lentils | 1 |

Table S1. Continued

| **Food group** | **Subgroup** | **Examples** | **# of foods** |
| --- | --- | --- | --- |
| Potatoes | Potatoes | Potatoes, regular and sweet; potato products | 9 |
| Fruits & juices | Apples | Apples | 1 |
|  | Pears | Pears | 1 |
|  | Plums | Plums | 1 |
|  | Sweet cherries | Sweet cherries | 1 |
|  | Apricots | Apricots | 1 |
|  | Peaches | Peaches | 1 |
|  | Strawberries | Strawberries (fresh and frozen) | 2 |
|  | Grapes | Grapes | 1 |
|  | Berries | Berries (blueberries, raspberries, blackberries) fresh & frozen | 5 |
|  | Dried fruits | Dried fruits (apples, raisins, plums, apricots) | 6 |
|  | Citrus fruits | Oranges and tangerines | 2 |
|  | Citrus fruits, other | Grapefruit, lemons, limes | 3 |
|  | Bananas | Bananas | 1 |
|  | Subtropical and tropical fruits | Mango, papaya, nectarines and other | 10 |
|  | Pineapple | Pineapple | 1 |
|  | Kiwi | Kiwi | 1 |
|  | Juices | Juices (apple, orange, grapefruit, carrot, tomato) | 6 |
| Nuts & seeds | Nuts | Nuts (hazelnuts, cashews, almonds, walnuts, coconut, pine seeds, pumpkin seeds, sunflower seeds, sesame seeds) | 17 |
|  | Poppy seeds | Poppy seeds | 1 |
| Meat and meat products | Pork | Pork and pork products; sausages cured and/or smoked | 12 |
|  | Beef | Beef (meat and tripe) | 6 |
|  | Meat, other | Lamb | 1 |
|  | Poultry | Poultry, whole and cuts; chicken meat products | 11 |
|  | Game | Game | 1 |
|  | Rabbits | Rabbit whole without head | 1 |
| Fish | Fish, total | Carp, trout, salmon, sardines, cod (natural and breaded), mackerel, shrimps, pangasius, cod liver | 21 |
| Fats and oils | Butter | Butter, not salted | 1 |
|  | Lard and bacon | Goose and pork lard, greaves, bacon | 5 |
|  | Vegetable edible fats and oils | Vegetable oil (olive, sunflower, rape seed, coconut), vegetable fat spread | 9 |
| Other foods: sugar, sweets and confectionary | Sugar | Sugar (white & brown), glucose, flavoured (vanilla) | 5 |
|  | Chocolate | Chocolate (white, dark and milk) | 4 |
|  | Cocoa | Cocoa powder | 1 |
|  | Cocoa products, other | Pralines and chocolate cookies | 3 |
|  | Confectionery products | Preserves (jam and marmalade) | 6 |
|  | Non-chocolate sweets | Sponge biscuits and pancakes | 2 |
|  | Honey | Bee honey | 1 |
|  | Yeast | Yeast | 1 |
|  | Salt | Salt (iodized) | 1 |
|  |  | Sum: | 330 |

Table S2. Amounts of single foods and their cost in the food basket for a family on minimum wage (MWFB) on a daily and monthly basis. RD, relative deviation from the reported intake of the corresponding category (Czech Statistical Office 2017).

| **Food group** | **Subgroup** | **Food name, Czech** | | **Food name, English** | | **Energy- adj. supply (g/day)** | **Weight raw (day)** | **Weight cat. in FB** | **RD** | **Weight edible (day)** | **Cost CZK (day)** | **Weight raw (month)** | **Cost CZK (month)** |
| --- | --- | --- | --- | --- | --- | --- | --- | --- | --- | --- | --- | --- | --- |
| Milk | Drinking milk cow | Mléko odtučněné | Milk, skimmed | | | 379 | 2213 | 2213 | +484% | 2213 | 30.8 | 67284 | 935.25 |
|  | Drinking milk goat | Mléko kozí | Goat milk | | | 0.6 | 0.6 | 0.6 | 0% | 0.6 | 0.1 | 19 | 1.69 |
| Milk products | Cheese | Sýr Lučina | Cream cheese | | | 83.6 | 26.4 | 26.4 | -68% | 26.4 | 5.3 | 803 | 160.25 |
|  | Curd and cottage cheese | Tvaroh polotučný | Curd, semifat | | | 27.6 | 27.6 | 27.6 | 0% | 27.6 | 2.1 | 840 | 63.54 |
|  | Other milk-based products | Podmáslí | Buttermilk | | | 212 | 107 | 107 | -49% | 107 | 2.8 | 3263 | 84.18 |
| Vegetables | Cucumber | Okurka | Cucumber | | | 40.2 | 20.6 | 20.6 | -49% | 19.9 | 1.0 | 625 | 30.01 |
|  | White pepper | Paprika bílá | White pepper | | | 36.4 | 36.4 | 36.4 | 0% | 29.9 | 2.0 | 1108 | 61.93 |
|  | Gherkins | Okurky nakládané | Gherkins | | | 18.8 | 18.8 | 18.8 | 0% | 29.4 | 1.2 | 573 | 35.41 |
|  | White cabbage | Zelí bílé | White cabbage | | | 49.6 | 200 | 200 | +303% | 184 | 2.2 | 6076 | 66.23 |
|  | Kale | Kapusta | Kale | | | 3.1 | 3.1 | 3.1 | 0% | 2.4 | 0.1 | 96 | 2.86 |
|  | Cauliflower | Květák | Cauliflower | | | 14.5 | 14.5 | 14.5 | 0% | 5.6 | 0.7 | 439 | 21.92 |
|  | White radish | Ředkev bílá | White radish | | | 13.2 | 13.2 | 13.2 | 0% | 46.5 | 0.8 | 401 | 24.66 |
|  | Yellow onion | Cibule žlutá | Yellow onion | | | 64.7 | 64.7 | 64.7 | 0% | 75.0 | 0.9 | 1967 | 27.35 |
|  | Garlic | Česnek | Garlic | | | 3.8 | 3.8 | 3.8 | 0% | 3.1 | 0.5 | 115 | 15.02 |
|  | Iceberg Lettuce | Ledový salát | Iceberg lettuce | | | 12.6 | 12.6 | 12.6 | 0% | 11.9 | 0.0 | 382 | 1.26 |
|  | Spinach frozen | Špenát mrazený | Spinach, frozen | | | 7.5 | 7.5 | 7.5 | 0% | 7.5 | 0.4 | 229 | 12.55 |
|  | Carrot | Mrkev | Carrots | | | 43.4 | 33.1 | 33.1 | -24% | 29.5 | 1.2 | 1007 | 37.64 |
|  | Parsley root | Petržel kořen | Parsley root | | | 6.3 | 6.3 | 6.3 | 0% | 6.5 | 0.5 | 191 | 14.20 |
|  | Celeriac root | Celer kořen | Celeriac root | | | 13.2 | 13.2 | 13.2 | 0% | 6.0 | 0.3 | 401 | 7.78 |
|  | Butter squash | Dýně Máslová | Butter squash | | | 51.5 | 51.5 | 51.5 | 0% | 48.2 | 1.8 | 1566 | 54.66 |
|  | Green peas, frozen | Hrášek zmrazený | Green peas, frozen | | | 4.4 | 4.4 | 4.4 | 0% | 3.5 | 0.3 | 134 | 9.13 |
|  | Green beans | Fazolky | Green beans | | | 1.9 | 1.9 | 1.9 | 0% | 1.3 | 0.2 | 57 | 5.71 |
|  | Champignion | Žampiony | Champignion | | | 17.6 | 5.6 | 5.6 | -68% | 5.7 | 0.7 | 169 | 20.11 |
|  | Broccoli | Brokolice | Broccoli | | | 70.4 | 61.3 | 70.4 | 0% | 42.2 | 3.3 | 1863 | 100.22 |
|  | Mustard, regular | Hořčice plnotučná | Mustard, regular | |  | | 9.1 |  |  | 14.5 | 0.3 | 276 | 9.40 |
| Pulses | Beans | Pečené fazole v rajčatové omáčce | Baked beans, white in tomato sauce | | | 6.3 | 2.0 | 6.3 | 0% | 1.5 | 0.2 | 60 | 4.66 |
|  |  | Fazole | Beans, dried | |  | | 4.3 |  |  | 16.9 | 0.4 | 131 | 12.49 |
|  | Peas | Hrách žlutý loupaný | Peas, yellow | | | 6.9 | 6.9 | 6.9 | 0% | 8.8 | 0.2 | 210 | 6.47 |
|  | Lentils | Čočka | Lentils | | | 4.4 | 4.4 | 4.4 | 0% | 16.9 | 0.3 | 134 | 10.40 |
| Potatoes | Potatoes | Brambory pozdní | Potatoes, regular | | | 434 | 324 | 400 | -8% | 256 | 4.8 | 9860 | 147.40 |
|  | Potato products | Hranolky smažené | French fries, frozen | |  | | 75.3 |  |  | 130 | 3.9 | 2288 | 118.77 |
| Fruits | Pears | Pear_raw | Pears, fresh | | | 21.6 | 21.6 | 21.6 | 0% | 19.5 | 1.1 | 657 | 32.79 |
|  | Plums | Švestky | Plums, fresh | | | 35.5 | 35.5 | 35.5 | 0% | 33.4 | 0.8 | 1079 | 23.09 |
|  | Sweet cherries | Třešně | Sweet cherries, fresh | | | 6.3 | 6.3 | 6.3 | 0% | 5.0 | 0.6 | 191 | 18.49 |
|  | Appricots | Meruňky | Appricots, fresh | | | 7.2 | 7.2 | 7.2 | 0% | 6.7 | 0.5 | 218 | 14.45 |

Table S2. Continued

| **Food group** | **Subgroup** | **Food name, Czech** | **Food name, English** | **Energy- adj. supply (g/day)** | **Weight raw (day)** | **Weight cat. in FB** | **RD** | **Weight edible (day)** | **Cost CZK (day)** | **Weight raw (month)** | **Cost CZK (month)** |
| --- | --- | --- | --- | --- | --- | --- | --- | --- | --- | --- | --- |
| Fruits | Strawberries | Jahody | Strawberries, fresh | 15.9 | 8.1 | 8.1 | -49% | 8.1 | 1.1 | 247 | 34.50 |
| (ctnd.) |  | Jahodový kompot | Strawberry compot | 3.8 | 3.8 | 3.8 | 0% | 3.8 | 0.4 | 115 | 11.15 |
|  | Blackberries | Ostružiny mrazené | Blackberries, frozen | 11.9 | 9.1 | 9.1 | -24% | 5.8 | 1.0 | 277 | 31.82 |
|  | Grapefruit | Grepy | Grapefruit, fresh | 26.6 | 26.6 | 26.6 | 0% | 13.3 | 1.1 | 808 | 34.66 |
|  | Oranges | Pomeranče | Oranges | 82.9 | 82.9 | 82.9 | 0% | 61.3 | 2.8 | 2519 | 85.41 |
|  | Bananas | Banány | Bananas | 67.3 | 34.4 | 34.4 | -49% | 22.0 | 1.3 | 1046 | 38.08 |
|  | Pineapple | Ananas | Pineapple,fresh | 9.1 | 9.1 | 9.1 | 0% | 4.6 | 0.5 | 277 | 15.78 |
|  | Kiwi | Kiwi | Kiwi | 5.9 | 5.9 | 5.9 | 0% | 4.5 | 0.7 | 180 | 19.98 |
| Nuts and | Nuts | Arašídy | Peanuts, peeled | 22.6 | 5.1 | 22.6 | 0% | 5.1 | 0.7 | 154 | 19.97 |
| seeds | Nuts | Sezamové semínka loupané | Sesame seeds, peeled |  | 17.6 |  |  | 17.6 | 2.8 | 534 | 84.86 |
|  | Poppy seeds | Mák | Poppy seeds | 2.7 | 2.7 | 2.7 | 0% | 2.7 | 0.3 | 82 | 9.20 |
| Cereals | Breakfast cereals | Kukuřičné lupínky | Corn flakes | 7.5 | 7.5 | 7.5 | 0% | 7.7 | 0.8 | 229 | 24.40 |
|  | Couscous | Kuskus | Couscous | 14.5 | 14.5 | 14.5 | 0% | 26.3 | 1.3 | 439 | 39.45 |
|  | Wheat flour | Pšeničná mouka polohrubá | Wheat flour, regular | 608 | 1251 | 1496 | +146% | 1251 | 18.0 | 38032 | 547.66 |
|  |  | Pšeničná mouka celozrnná | Wheat flour, wholegrain | | 241 |  |  | 241 | 8.4 | 7317 | 255.38 |
|  |  | Semolinová mouka | Wheat flour, semolina | | 4.7 |  |  | 51.6 | 0.1 | 143 | 4.54 |
|  | Rye flour | Žitná mouka celozrnná | Rye flour, wholegrain | 57.8 | 57.8 | 57.8 | 0% | 57.8 | 2.6 | 1757 | 78.02 |
|  | Barley | Kroupy ječné | Barley groats | 11.9 | 11.9 | 11.9 | 0% | 35.1 | 0.3 | 363 | 9.36 |
|  | Spelt flour | Špaldová mouka | Spelt flour | 3.8 | 3.8 | 3.8 | 0% | 3.8 | 0.3 | 115 | 8.01 |
|  | White bread | Strouhanka | Bread crumbs | 49.6 | 49.6 | 49.6 | 0% | 49.6 | 1.9 | 1509 | 57.04 |
|  | Eggless pasta | Těstoviny bezvaječné | Eggless pasta | 44.6 | 44.6 | 44.6 | 0% | 116 | 1.5 | 1356 | 45.84 |
| Meat | Pork | Jitrnice | White meat pudding | 269 | 43.8 | 43.8 | -84% | 43.8 | 4.4 | 1332 | 134.54 |
|  | Poultry | Kuřecí játra | Chicken liver | 168 | 186 | 186 | 10% | 140 | 12.1 | 5646 | 366.43 |
|  | Red meat, other | Jehněčí filety | Lamb fillets | 2.5 | 1.9 | 1.9 | -24% | 1.8 | 1.1 | 58 | 32.03 |
|  | Game | Jelení medailonky | Deer medaillons | 5.7 | 4.3 | 4.3 | -24% | 4.3 | 1.1 | 131 | 32.69 |
|  | Rabbit | Králík celý, bez hlavy | Rabbit, whole | 5.0 | 5.0 | 5.0 | 0% | 3.9 | 1.1 | 153 | 33.38 |
| Fish | Fish | Krabí tyčinky surimi | Crabstics surimi | 32.0 | 1.5 | 26.5 | -17% | 2.3 | 0.2 | 44 | 6.92 |
|  | Fish | Matjesy v oleji | Herring in oil |  | 25.1 |  |  | 25.1 | 4.3 | 762 | 131.14 |
| Fats and | Butter | Máslo nesolené | Butter unsalted | 33.9 | 9.8 | 9.8 | -71% | 9.8 | 2.1 | 297 | 62.82 |
| oils | Lard and bacon | Sádlo vepřové | Pork lard | 28.3 | 71.8 | 71.8 | +154% | 71.8 | 5.9 | 2184 | 178.64 |
|  | Vegetable oils | Tuk rostlinný roztíratelný | Vegetable fat spread | 108 | 179 | 207 | +91% | 179 | 17.9 | 5449 | 542.74 |
|  |  | Olej řepkový | Rape seed oil |  | 7.4 |  |  | 7.4 | 0.3 | 224 | 10.07 |
|  |  | Olej slunečnicový | Sunflower oil |  | 20.0 |  |  | 20.0 | 0.9 | 609 | 26.11 |
| Other | Sugar | Cukr bílý | White sugar | 214 | 59.5 | 59.5 | -72% | 59.5 | 1.3 | 1809 | 39.62 |
|  | Cocoa | Kakaový prášek | Cocoa powder | 3.1 | 3.1 | 3.1 | 0% | 3.1 | 1.4 | 96 | 42.88 |
|  | Non-chocolate sweets | Piškoty | Sponge biscuit | 18.8 | 14.4 | 14.4 | -24% | 14.4 | 1.5 | 438 | 47.04 |
|  | Honey | Včelí med | Bee honey | 5.7 | 5.7 | 5.7 | 0% | 5.7 | 1.1 | 172 | 34.35 |
|  | Yeast | Droždí | Yeast | 7.4 | 7.4 | 7.4 | 0% | 7.4 | 0.7 | 225 | 22.00 |
|  | Salt | Sůl | Salt | 35.8 | 12.2 | 12.2 | -66% | 12.3 | 0.3 | 371 | 10.40 |
|  |  |  | **Sums:** | **7484** | **5994** | **5994** |  | **6043** | **177.7** | **182213** | **5401** |
